# Schismogenesis in anxiety spectrum disorders: a biopsychosocial perspective

**DOI:** 10.3389/fpsyt.2025.1667066

**Published:** 2026-01-14

**Authors:** Mauro García-Toro, Rocío Gómez-Juanes

**Affiliations:** 1 Research Network on Chronicity, Primary Care and Health Promotion (RICAPPS), Carlos III Health Institute, Madrid, Spain; 2 University Institute of Health Science Research (IUNICS), University of the Balearic Islands, Palma, Spain; 3 Health Research Institute of the Balearic Islands (IdISBa), Palma, Spain; 4 Department of Medicine, University of the Balearic Islands, Palma, Spain

**Keywords:** anxiety disorders, complexity, dissociation, models, biopsychosocial, systems thinking

## Abstract

Anxiety spectrum disorders (ASDs) often have an unsatisfactory prognosis, suggesting the opportunity for complementary explanatory frameworks to advance their therapeutics. This text advocates for a framework rooted in cybernetics and complex systems theory, which views the mind, brain, and social networks as deeply interdependent systems. A characteristic feature of such systems is the operation of similar organizational principles and laws across different levels of analysis, a phenomenon termed isomorphism. Thus, the mind, brain, and social systems operate under isomorphic principles, requiring a critical balance between stability (homeostasis) and the capacity for change (homeodynamics) to successfully adapt to environmental perturbations. From this perspective, the central challenge under stress is to prevent excessive fragmentation and functional dissociation, a process termed schismogenesis in cybernetics. ASDs are, therefore, reconceptualized as biopsychosocial dissociations stemming from a schismogenic mechanism. This framework posits that mental health is contingent upon maintaining a dynamic equilibrium between connectedness and independence across social, mental, and neural levels. It also suggests that any intervention promoting reintegration can be therapeutic when dissociation occurs. While single-component psychosocial approaches may suffice for mild cases of ASDs, the ideal therapeutic plan for severe or refractory cases should be rapidly implemented, personalized, multicomponent and synchronous.

## 1 Introduction

Scientific thought has alternated between linear and circular paradigms over the past centuries. While medicine’s Hippocratic origins were more circular, the linear approach has predominated in recent decades (1). These terms represent a fundamental dichotomy in scientific perspectives (2).

The linear paradigm (analytical, reductionistic, deterministic) focuses on identifying a primary cause through direct, unidirectional cause-and-effect relationships. It decomposes systems into their smallest components, assuming events are predetermined and predictable. This approach favors quantification, statistical analysis, and conceives processes as a linear sequence. It forms the basis of the classical scientific method, promoting disciplinary knowledge and specific therapies targeting disease causes (3).

Conversely, the circular paradigm (systemic, holistic, nonlinear, dynamic, complex) views living organisms as open systems with self-organization (4). It acknowledges that phenomena result from multiple interconnected factors and feedback loops, making cause and effect difficult to delineate (5). This perspective emphasizes understanding systems as integrated wholes, where interactions are crucial (6). It recognizes non-linearity, inherent uncertainty, and emergent properties, valuing qualitative understanding and dynamic, recursive processes (6, 7). Treatments within this framework aim to rectify systemic imbalances. While not rejecting the scientific method, it expands it to address complexity and interconnectedness, promoting transdisciplinary approaches (3, 8, 9).

Historically, the influence of these paradigms in medicine coincided in the work of Louis Pasteur (linear, germ theory) and Claude Bernard (circular, systemic balance). Pasteur’s linear approach gained prominence due to its immediate practical results, further solidified by antibiotic discoveries. However, the circular paradigm, particularly in mental health, remains a fertile framework (10–12).

The General Systems Theory and the biopsychosocial model provide strong support for the circular approach (13, 14). General Systems Theory advocates for transdisciplinarity to foster unity and communication across scientific fields (3, 15). The biopsychosocial model, built on these principles, posits that biological, psychological, and social factors interact circularly to influence health (4, 8). Although a foundational framework, the biopsychosocial model has been criticized for its incomplete development in fully understanding the causal and therapeutic mechanisms of mental health conditions, prompting the need for new proposals to enhance its utility (16–18).

Ultimately, the choice of paradigm depends on a system’s complexity and the specific research questions. While linear thinking suits simple relationships, the circular paradigm offers a more appropriate understanding of complex, dynamic systems (13, 15). It is crucial to highlight that these paradigms are not mutually exclusive. A comprehensive understanding often necessitates their combination, as they are complementary rather than alternative problem-solving approaches that can be applied at different levels of analysis (2, 16).

## 2 Schismogenesis: an integrative transdisciplinary cybernetic concept applied to the biopsychosocial model

Mind, brain, and social networks are interdependent complex systems (15, 19). A complex system operates optimally when there is a balance among its components, and the flow of energy and information occurs without significant blockages (3). However, the mechanisms underlying imbalances between connection and differentiation remain to be fully understood (9, 15).

A complex system can exhibit three types of responses, contingent upon the ratio of stimulatory to inhibitory interactions (**Table 1**). Homeostasis focuses on maintaining a constant internal state. It involves regulatory mechanisms based on negative feedback, primarily due to the predominance of inhibitory interactions, which counteract perturbations and restore equilibrium (10, 11). Homeodynamics refers to a system’s capacity to dampen disturbances without destabilization, while concurrently utilizing them for adaptive change (20). This necessitates a sufficient balance between positive and negative feedback to adaptively reconcile tendencies towards homeostasis and homeodynamics or morphogenesis. The third possibility arises when stimulatory interactions initiated within a part of the system are disproportionately greater than inhibitory ones. Positive feedback loops then emerge and amplify without adequate moderation by negative feedback, leading to system destabilization and fragmentation, a process known as schismogenesis (5, 21). The term “schismogenesis” was coined by Gregory Bateson, a biologist, anthropologist and cyberneticist known for his highly creative, interdisciplinary approaches (22). As such, schismogenesis is a construct from cybernetics used to explain a type of runaway feedback process in the dynamics of relationships (21, 23). In what follows, we explore how this construct can be applied from a biopsychosocial perspective to elucidate the mechanism underlying the genesis of mental disorders.

**Table 1:** Core concepts in cybernetics and dynamics complex systems theory.

Concept	Definition in complex systems	Role of negative feedback	Role of positive feedback	System-level outcome
Homeostasis	The system maintains internal stability despite external perturbations.	Dominant; stabilizes the system by counteracting deviations.	Minimal; tightly regulated to avoid runaway effects.	Stable, regulated equilibrium with low variability.
Homeodynamics	The system sustains functional organization through dynamic adaptation rather than fixed steady states.	Help modulate fluctuations without eliminating them; supports flexible stability.	Supports adaptive change by amplifying signals needed for reorganization.	Dynamic equilibrium with controlled variability and adaptive capacity.
Schismogenesis	Escalating divergence between system components driven by unmoderated feedback interactions, leading to fragmentation.	Insufficient; fails to compensate for overstimulation between subsystems.	Dominant; reciprocal amplification drives escalating separation.	System destabilization, polarization, or fragmentation into competing subsystems.
Isomorphism	Structural or functional correspondence across different system levels (e.g., neural, mental, social).	Ensures parallel stabilizing mechanisms across levels.	Mirrors amplification dynamics across levels (e.g., social → mental → neural).	Cross-level patterning: similar dynamics appear at different scales of the system.
Dissociation	Breakdown of coordination among subsystems due to disrupted feedback loops.	Impaired; fails to maintain integration.	May become chaotic or unregulated, contributing to subsystem isolation.	Loss of coherence; subsystems operate asynchronously or independently.

**Figure 1:**
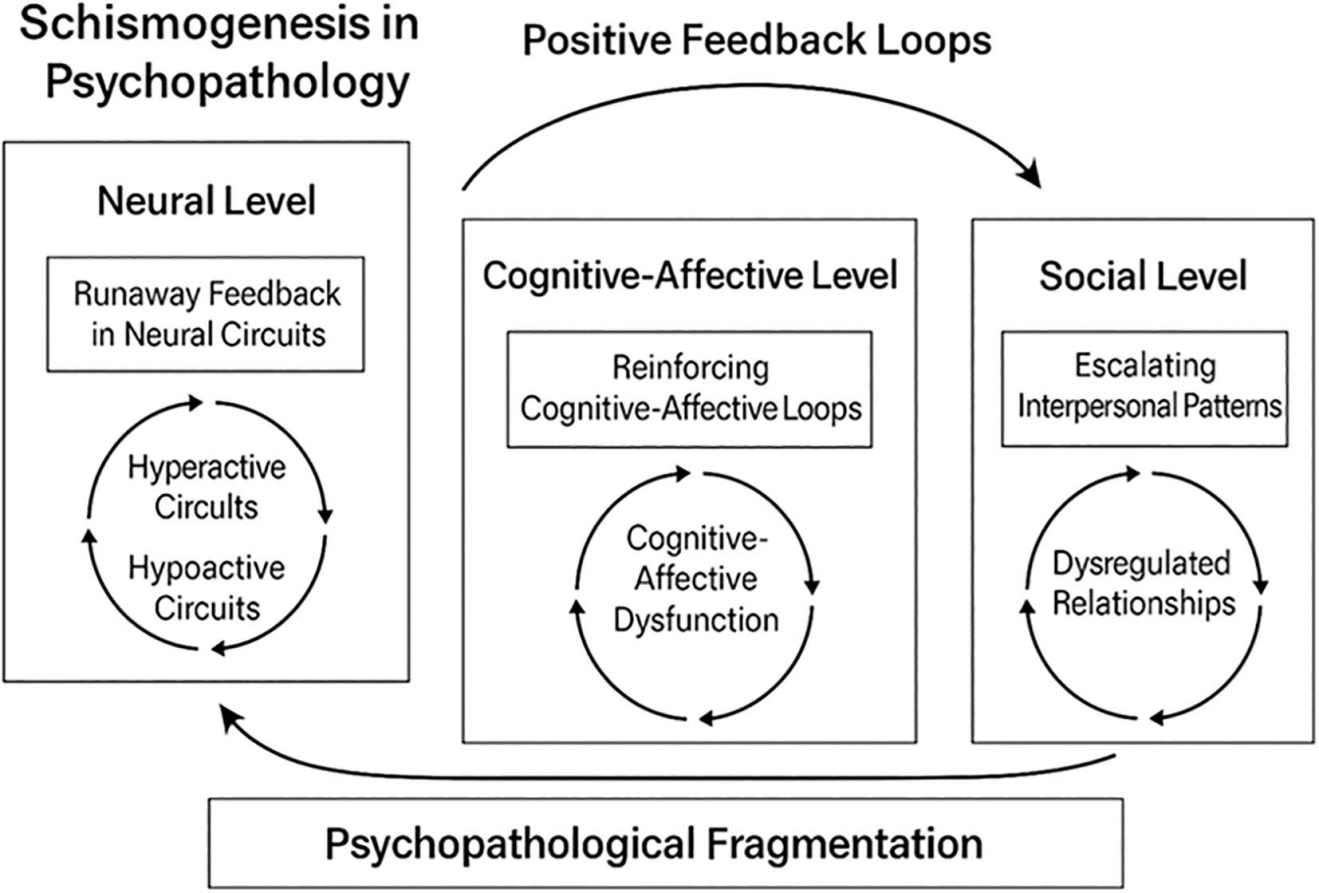
Schismogenesis as a key construct in psychopathology.

When mind, brain, and social systems are overloaded beyond the threshold of their inhibitory functional reserve, they enter a reverberant dynamic. This leads to the autonomization of system parts due to complementary schismogenesis, resulting in dissociation. Furthermore, this functional fragmentation gives rise to distinct neural, mental, and social states: hyperactive domains that disregards the regulation of the rest of the system, and hypoactive domains that attempts to counteract overactivity. Although these domains may overlap and intermingle at the brain level, neuroimaging studies have identified such patterns in various mental disorders, linking hyperactivity to positive symptoms and hypoactivity to negative symptoms (22). At a mental level, a portion of the symptom network becomes self-sustaining and self-reinforcing, requiring the inhibition or hypoactivation of the remaining network to prevent the progression of the positive feedback loop (21). This implies a mental dissociation. At a social level, when an individual experiences an overload, for example, due to a significant loss that cannot be managed in a coordinated manner with their other most significant relationships, they may dissociate from the social networks with which they previously interacted. This results in emotional and communicative isolation, although in some cases, a few very close ties may be preserved (24) (**Figure 1**).

## 3 Generic hypotheses derived from assuming schismogenesis as a biopsychosocial origin of mental disorders

Biological, psychological, and social perspectives represent distinct levels of analysis for understanding mental disorders and behavior (19). None of these levels is inherently more or less important (ontologically primary or secondary), although for each patient at any given time, one or another may be more relevant for therapeutic intervention (25). Furthermore, they are inextricably linked; any occurrence at the biological level invariably has a simultaneous impact at the psychosocial level, and vice versa (13). Consequently, it is posited that the schismogenic dissociation underlying mental disorders is isomorphic across social, mental, and neural networks (15). This premise gives rise to several hypotheses, each currently exhibiting variable empirical support.

No single cause is either necessary or sufficient for the onset of any mental disorder. Numerous predisposing factors increase susceptibility by altering the excitatory/inhibitory balance within brain, mental, and social networks. Similarly, various factors act as precipitants or stressors by sufficiently activating these networks to expose an insufficiency of inhibitory modulation (26). When this stress-vulnerability imbalance permits uncontrolled positive feedback within brain, mental, and social networks, it initiates a process of schismogenesis. Pathogenesis, therefore, is rooted in the process of complementary schismogenesis. It involves a dynamic change that undergoes amplification within a system, ultimately resulting in its functional fragmentation due to insufficient inhibitory control (21).

A complementary schismogenic process implies the emergence of functionally distinct domains exhibiting reciprocally conditioned levels of activation. The clinical expression will vary depending on the predominant localization of rigidly hyperactivated and rigidly hypoactivated areas within mental, brain, and social networks. While every individual with psychopathology presents distinct symptomatology, grouping them into diagnostic categories may be useful for optimizing treatment focus, while acknowledging the full spectrum of intermediate and overlapping presentations (27–30). Diagnosis should be personalized and based on measures of cerebral, mental, and social dysconnectivity. These can be quantified through neuroimaging techniques, standardized scales, and other methods (31). Furthermore, these transdiagnostic dysconnectivity indices would aid in monitoring therapeutic response and evolution (32).

The primary therapeutic approach for an individual with a mental disorder is to assist in reducing the escalation of biological or psychosocial stress that may have precipitated schismogenesis. Through this intervention, some patients may achieve reintegration. Unfortunately, this is not always feasible. Many biological stressors are not easily identified and neutralized (e.g., comorbid illnesses, substance use involving toxins, drugs, or pharmaceuticals) (21). The same applies to psychosocial stressors (e.g., loss of significant relationships).

As a second step, it is imperative to assist the patient’s reintegration at a biopsychosocial level. However, it is crucial to remember that systems cannot be directed, only perturbed. For example, focally perturbing the activation levels of the networks (stimulating what is rigidly inhibited or inhibiting what is rigidly stimulated) in a manner that favors re-equilibrium and reintegration. This idea is highly compatible with the proposals of the neurobiologist Humberto Maturana (35). As psychotherapists that followed his teachings noted, treatment does not directly “change” the patient. Instead, it functions as a catalytic perturbation, which the patient’s system processes to generate its own internal reorganization. This shifts the therapeutic goal from “curing” to supporting the patient’s inherent capacity for self-creation and transformation (35).

Prevention is also contingent upon the equilibrium between stress and inhibitory neural, cognitive, and social control mechanisms. Individual responsibility regarding one’s mental health is paramount in this context. However, a highly pertinent, albeit underdeveloped, community-based intervention approach also exists. For instance, evidence indicates that the Western lifestyle elevates the risk of some mental disorders (33). As an example, it might be advantageous to establish legal regulations to combat marginalization, as well as to help people maintain healthy dietary patterns, active behaviors, and sufficient sleep duration, especially within vulnerable populations (25, 34).

## 4 Anxiety spectrum disorders conceptualized as biopsychosocial dissociation resulting from schismogenesis

ASDs encompass a heterogeneous array of mental disorders distinguished by excessive and enduring fear, worry, and associated behavioral disturbances (35, 36). While once considered less debilitating than major depressive disorder, ASDs are now recognized for their substantial global prevalence and significant contribution to disease burden. Epidemiological data indicate that lifetime prevalence rates for any anxiety disorder range from 10% to over 30% in different populations, often surpassing those of other mental health conditions (37). This high prevalence translates into considerable disability, impacting multiple life domains, including occupational functioning, social relationships, and overall quality of life (38). Furthermore, ASDs are frequently comorbid with other physical and mental health conditions, exacerbating their disabling effects and complicating treatment trajectories. The economic impact of ASDs is also substantial, driven by healthcare utilization, productivity loss, and informal caregiving burdens (39). Consequently, understanding and addressing the prevalence and disabling sequelae of ASDs remain a critical public health priority.

A prominent category within this spectrum is Generalized Anxiety Disorder (GAD), wherein individuals experience chronic and excessive worry regarding a variety of events or activities (40). This worry is often difficult to control and is associated with physical symptoms such as restlessness, fatigue, and muscle tension (41). Panic Disorder (PD) constitutes another significant anxiety disorder characterized by recurrent and unexpected panic attacks, which are defined as sudden surges of intense fear or discomfort that peak within minutes and encompass both physical and cognitive symptoms (35). Individuals with PD frequently develop anticipatory anxiety and a fear of future attacks, sometimes leading to agoraphobia, a marked fear or avoidance of situations where escape might be difficult or where help may not be readily available (42).

Social Anxiety Disorder (SAD), also known as social phobia, involves a marked fear or anxiety about one or more social situations in which the individual is exposed to possible scrutiny by others (35). This fear often revolves around negative evaluation, embarrassment, or rejection. Specific phobias are characterized by marked fear or anxiety about a specific object or situation (e.g., spiders, heights, flying…). Exposure to the phobic stimulus almost invariably provokes an immediate fear or anxiety response (43). Obsessive-Compulsive Disorder (OCD) is now classified separately from the anxiety disorders in DSM-5 but is considered related due to its significant anxiety component. It involves the presence of obsessions (recurrent and persistent thoughts, urges, or images that are experienced as intrusive and unwanted) and/or compulsions (repetitive behaviors or mental acts that an individual feels driven to perform in response to an obsession or according to rules that must be applied rigidly) (35). Posttraumatic Stress Disorder (PTSD) is another disorder now classified separately, within a trauma- and stressor-related disorders category. It develops after exposure to a traumatic event and is characterized by intrusive memories, avoidance of trauma-related stimuli, negative alterations in cognition and mood, and marked alterations in arousal and reactivity (44).

In psychopathology, the term “dissociation” has historically encompassed diverse meanings (45). Current international classifications define it as a disruption or disconnection in the integrative functions of consciousness, memory, identity, emotion, perception, body representation, motor control, and behavior (35). Dissociation is conceptualized as a continuum, ranging from normative, everyday experiences (e.g., daydreaming, absorption in a book) to severe and pathological forms observed in dissociative disorders (46, 47).

It is widely accepted that in traumatic situations, dissociation can function as a defense mechanism, enabling individuals to psychologically distance themselves from overwhelming experiences (48). Dissociative phenomena can also manifest in the context of other mental disorders, though they may be underrecognized (46, 47). This disconnection varies in modality and intensity and can be present where significant psychological stress plays an etiological role or exacerbates symptomatology (49). For instance, in anxiety disorders, individuals may report emotional detachment or numbing, which are conceptualized as forms of dissociation (50). Furthermore, rumination, a common feature in anxiety, can be interpreted as a form of dissociation from present-moment awareness due to preoccupation with intrusive thoughts (51).

Post-traumatic stress disorder (PTSD) has been proposed to have a dissociative origin in a subset of cases (52). Trauma theory offers a mechanistic explanation for this dissociation: an uncontrolled positive feedback loop (a vicious cycle) that continues until compensatory inhibition from other system components can halt its progression (21, 53). This process is analogous to complementary schismogenesis, implying the dissociation of the social system, mind, and brain (in interaction with the rest of the body) into two reciprocally conditioned, unstable domains: a hyperactivated domain and a hypoactivated domain.

However, trauma alone does not fully explain the onset of PTSD. Other factors are more determinant, including social vulnerability (e.g., absence of meaningful relationships to stabilize tensional overload), psychological vulnerability (e.g., insufficient inhibitory self-control to stabilize the mind), or cerebral vulnerability (e.g., inadequate inhibitory neuronal interactions to stabilize the nervous system). Trauma theory suggests that PTSD is a pathological response to a traumatic experience that overwhelms an individual’s processing capacity, leading to persistent symptoms of re-experiencing, avoidance, hyperarousal, and negative alterations in cognition and mood. Understanding this theory is crucial for the effective diagnosis and treatment of PTSD. Its relevance could be posited for other ASDs, even in the absence of clear traumatic triggers (54).

In ASDs, a dissociation of cerebral functioning may occur. Areas associated with threat detection and emotional response (e.g., the amygdala) often exhibit hyperactivation, while regions responsible for emotional regulation, contextual memory, and danger assessment (e.g., the prefrontal cortex and hippocampus) frequently show hypoactivation (45, 55). This dysfunctional communication between brain regions underlies many characteristic anxiety symptoms that intrude into consciousness involuntarily, thereby depleting cognitive resources and leading to cognitive blocking (23). At a psychosocial level, this can manifest as blockage, isolation, and relational rigidity (21). Consequently, dissociation in ASDs can significantly impede healthy social functioning by altering the perception of self, others, and the world, thus hindering the formation and maintenance of meaningful relationships, effective communication, and full participation in social life (45, 54–56).

Scientific literature communicates findings consistent with post-schismogenesis self-maintaining dissociation as a plausible explanatory mechanism for Anxiety Spectrum Disorders (ASDs) across the biological, psychological, and social levels. Neuroimaging evidence further suggests that ASDs feature hyperconnectivity within salience-amygdala circuits and hypoconnectivity within prefrontal regulatory networks. This pattern precisely reflects the kind of complementary hyper-/hypo-active dissociative pattern predicted by the Schismogenesis Model (57–59). Sustained overactivation of neural networks in anxiety disorders has been suggested to induce excitotoxicity, neuroinflammation, and oxidative stress, although discordant interpretations exist (60–62). These processes increase neuronal excitability, exacerbate anxiety symptoms, and heighten vulnerability to comorbid mental disorders (63). Other neuroimaging and molecular studies also demonstrate that chronic stress and anxiety are associated with hyperactivity in limbic and prefrontal circuits, increased inflammatory markers, and oxidative damage to neural tissue (62, 64–66).

**Figure 2:**
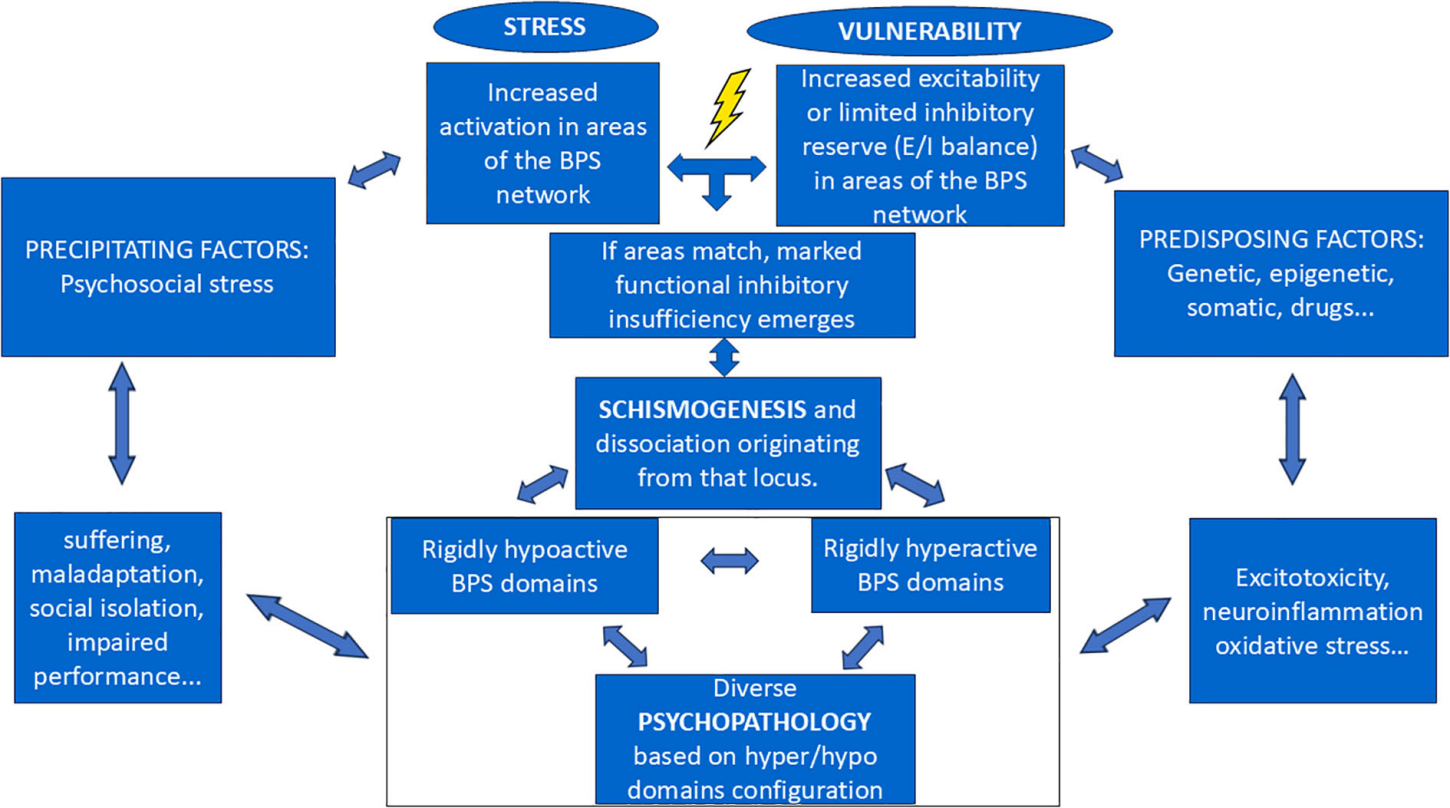
Schismogenesis offers a biopsychosocial (BPS) mechanism that links stress and vulnerability to the emergence of psychopathology.

On the psychosocial level, anxious psychopathology is consistently linked to increased suffering, maladaptation, social isolation, and impaired performance (65, 67, 68). These factors contribute to elevated psychosocial stress, which perpetuates and reinforces anxiety disorders, thereby establishing a circular pathogenic mechanism (69–71) (**Figure 2**). Anxiety disorders are associated with reduced interpersonal synchrony, social withdrawal, and disruptions in affiliative signaling — social manifestations of a systemic dissociation (72, 73).

## 5 Contributions of schismogenesis to the comprehensive etiopathogenesis of ASDs

**Figure 3:**
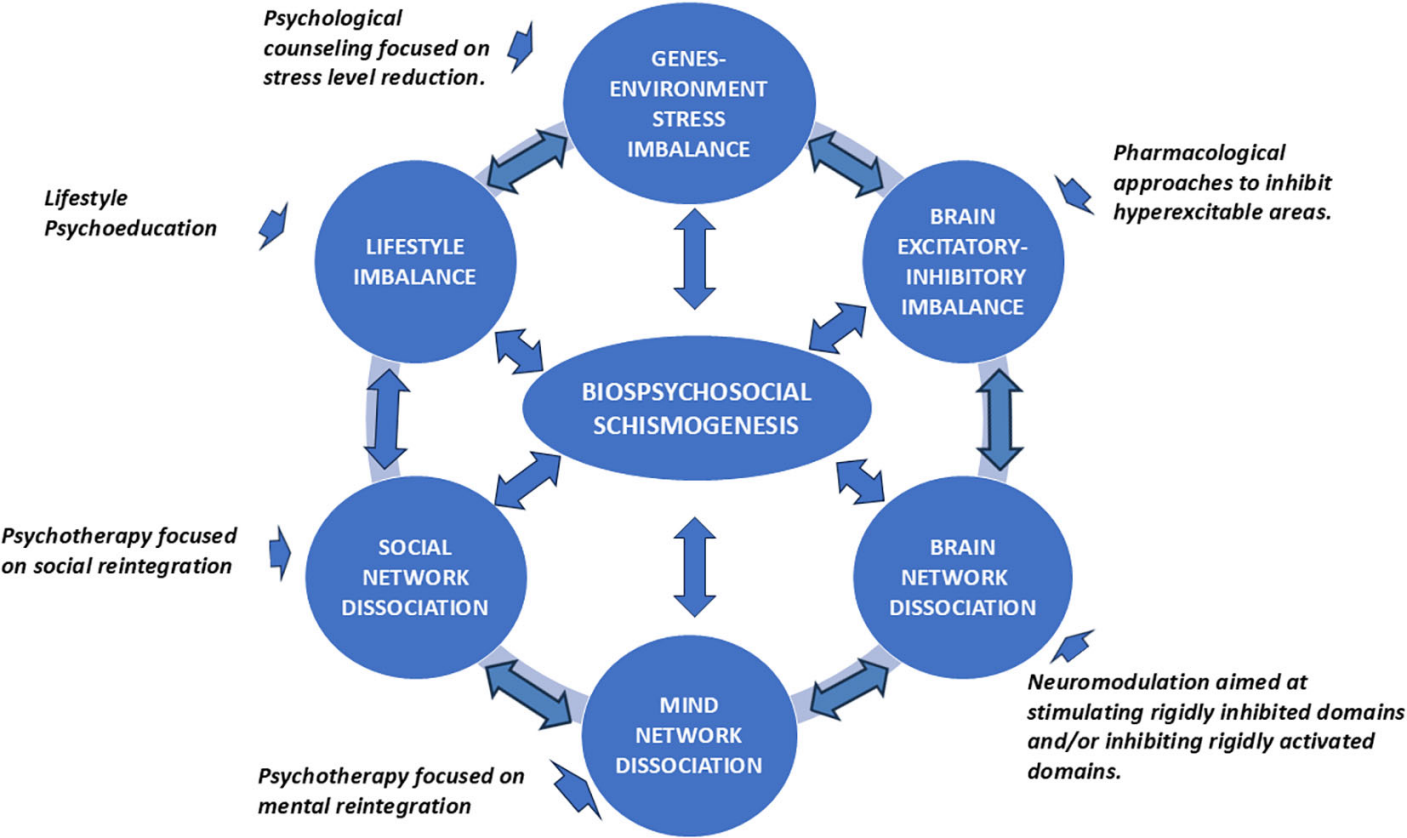
Circular etiopathogenesis and treatment of anxiety spectrum disorders (ASDs) within the conceptual framework of biopsychosocial schismogenesis. Etiopathogenesis can be conceptualized as a self-sustaining multilevel vicious cycle that may intensify over time. Fortunately, the inertia of this dynamic can be interrupted by various therapeutic approaches, offering improved outcomes when applied promptly and synergistically (21).

ASDs, viewed through the biopsychosocial paradigm and circular causality, can be understood as the dynamic, reciprocal, and multilevel interaction of six significant constructs in their manifestation, extensively supported by research (**Figure 3**). Each of these six constructs can be considered related to biopsychosocial schismogenesis and a pathway for therapeutic intervention, exerting an immediate influence on the five others (74). We refer to Mind Network Dissociation, Social Network Dissociation, Brain Network Dissociation, Lifestyle Imbalance, Gene-Stress Imbalance and Brain Excitatory-Inhibitory Imbalance.

Genes-Environment Stress Imbalance in ASDs refers to the intricate interplay between an individual’s genetic predispositions and encountered environmental stressors (75, 76). This dynamic interaction, rather than genes or stress alone, contributes to the development and manifestation of these disorders.

The concept of excitatory/inhibitory (E/I) imbalance in the brain refers to a disruption in the proportion between excitatory and inhibitory neurotransmission. This imbalance has been increasingly implicated in the pathophysiology of various neuropsychiatric disorders (77). In ASDs, evidence suggests a dysfunction in the neural circuits that regulate fear and anxiety, where an increase in excitation or a decrease in inhibition may lead to hyperreactivity to perceived threats and a difficulty in regulating emotional responses (78–80).

Brain network dissociation in ASDs refers to disruptions or alterations in the coordinated activity and communication between different functional brain networks (31). Instead of working harmoniously, these networks may show abnormal levels of connectivity (either hyper- or hypo-connectivity) or a lack of the typical integration that supports healthy emotional regulation and cognitive processing (81, 82). In this context, the amygdala and prefrontal cortex are highly significant structures (78, 81).

Network analysis views mental disorders as complex systems of interacting symptoms (83, 84). In this framework, symptoms are not just passive indicators of a disorder but actively influence each other, contributing to the maintenance and development of the condition (26). Mind network dissociation is often viewed as a defense mechanism against overwhelming stress or trauma (23). It can involve a disconnection from the present moment, emotional numbing, and altered perceptions (50, 85–88).

Social network dissociation in the context of ASDs refers to a individual sense of detachment or disconnection specifically within the realm of social interactions (89). Social disconnection is a common and pernicious feature of ASDs yet is usually insufficiently addressed by our best available treatments (90, 91).

Addressing lifestyle imbalances is a fundamental aspect of the management and prevention of ASDs. Promoting healthy habits in sleep, nutrition, physical activity and stress management can enhance resilience, improve emotional regulation, and reduce vulnerability. Frequently, therapeutic interventions for ASDs incorporate recommendations and strategies to foster a more balanced lifestyle as a key component of treatment (92).

## 6 Discussion

Treatments for ASDs may be effective by facilitating the reintegration of dissociation, potentially through systemic stress reduction. Chronic stress dysregulates the hypothalamic-pituitary-adrenal (HPA) axis, leading to hypercortisolemia and structural changes in brain regions vital for emotional regulation, such as the amygdala and prefrontal cortex (34, 93). Psychologically, stress exacerbates negative cognitive patterns, while socially, it can strain relationships (94). Reducing stress can interrupt maladaptive thought processes by fostering control and improve social engagement by strengthening support networks (24, 95, 96).

Given the interconnectedness of biological, psychological, and social factors, interventions at one level can impact others. Combined treatments, like psychotherapy and pharmacotherapy, show superior efficacy in managing ASDs compared to monotherapy (36, 86). This aligns with focusing on the interpersonal domain for comprehensive and personalized care (33). Furthermore, lifestyle modifications, including regular physical activity, a balanced diet, and adequate sleep, are promising adjuncts to traditional treatments, significantly reducing anxiety (97–100).

Timely intervention is crucial for effective Social Anxiety Disorder (SAD) treatment, leading to better resolution and preventing recurrence (101, 102). While definitive evidence is challenging to obtain, it is increasingly clear that the neurobiological underpinnings of SAD, such as heightened amygdala activity and disrupted prefrontal cortex regulation, may become more ingrained over time (84, 86, 103). This entrenchment can reduce neural plasticity and treatment responsiveness, as well as dissociate mental and social networks (83–86, 104, 105). Serotonergic medications may complement psychosocial interventions by enhancing serotonin in the non-dominant hemisphere, which is often relatively hyperactive in SAD (77). This could dampen negative emotional processing and facilitate regulatory functions in the dominant hemisphere (21, 106). Therefore, maximizing the synergistic effects of combined SAD treatments is more likely achieved through early initiation.

Effective management of ASDs requires personalized biopsychosocial interventions based on comprehensive individual assessments (106). These assessments should encompass biological, psychological, and social factors to inform tailored therapeutic plans (107). Optimizing psychosocial interventions involves evaluating individual characteristics like anxiety subtype, symptom severity, comorbidities, and cognitive patterns (108). At the biological level, non-invasive brain stimulation techniques such as transcranial magnetic stimulation (TMS) and transcranial direct current stimulation (tDCS) show promise (109, 110). These techniques modulate neural activity in anxiety-related brain regions, including the amygdala and prefrontal cortex. Repetitive TMS has demonstrated efficacy in reducing anxiety symptoms and improving quality of life in treatment-resistant patients, though optimal parameters and mechanisms require further research (110, 111). As previously indicated, evidence suggests that individuals with ASDs frequently exhibit increased right (non-dominant) hemisphere activity (106, 112). This phenomenon, also observed in major depression, is implicated in the processing of negative emotions, threat detection, and visceral responses (113). Conversely, the left (dominant) hemisphere is more associated with positive affect and cognitive control (114). Effective neuromodulation strategies generally involve inhibitory action on the right hemisphere and stimulatory action on the left hemisphere (110, 115). However, heterogeneity in research findings underscores the need for further investigation, particularly into individual brain activation patterns to personalize treatment (109, 110, 116).

To overcome the self-perpetuating inertia of dynamic schismogenesis, synchronous combined treatments might be more effective. For instance, neuromodulatory interventions hold promises for enhancing the efficacy of psychotherapeutic treatments, including those for phobic and obsessive-compulsive disorder (OCD) (109, 110, 116). As previously suggested, neuromodulation techniques can directly influence neural circuits involved in fear and anxiety, such as the amygdala and prefrontal cortex (103, 112). For example, low-frequency repetitive transcranial magnetic stimulation (rTMS) can inhibit overactive regions like the amygdala, potentially reducing fear response intensity during exposure therapy (103, 109, 110). Conversely, high-frequency rTMS or anodal transcranial direct current stimulation (tDCS) over the prefrontal cortex can enhance cognitive control and emotional regulation, facilitating the processing of safety signals during exposure and promoting the consolidation of new, non-fearful associations (113, 114, 117). By leveraging the immediate neurobiological effects of neuromodulation to augment the psychological processes of fear extinction and cognitive restructuring, synchronous combined treatments may lead to more rapid, robust, and enduring outcomes for individuals with phobias (104). When biological and psychological therapies are synchronized and coordinated, the synergistic effects can be sufficiently enhanced to overcome the inertia of dynamic schismogenic dysfunction, thereby facilitating reintegration (21).

As suggested in the preceding paragraph, the induction of anxious psychopathology can enhance the efficacy of concurrent treatments, as exemplified also by Eye Movement Desensitization and Reprocessing (EMDR). EMDR has emerged as a promising therapeutic approach for individuals with ASDs, building on its successful application in trauma-related anxiety, where adverse life experiences and maladaptive cognitions are often etiologically significant (118). Research suggests EMDR reduces anxiety symptoms by processing and integrating distressing memories. Through bilateral stimulation (e.g., eye movements) while recalling difficult experiences, EMDR aims to lessen their emotional impact. While mechanisms are still being explored, bilateral stimulation may help to reintegrate reverberant mind and brain networks. Bilateral movements activate prefrontal areas, and this could lead to weakening rigidly activated neural networks and promoting their integration (119, 120). For example, studies on Generalized Anxiety Disorder (GAD) demonstrate EMDR’s effectiveness in improving worry, anxiety levels, and associated symptoms (118, 119). Its efficacy in ASDs may stem from its ability to target underlying emotional and cognitive processes that maintain anxiety, fostering a more balanced and adaptive perspective and reducing vulnerability to triggers. The rationale for intentionally triggering anxiety symptoms suggests that a heightened anxious state may increase neural circuit responsiveness to EMDR intervention (121).

### 6.1 Studies supporting the schismogenesis hypothesis or its components

Although the specific term schismogenesis has been rarely used in empirical psychopathology research, key components of the proposed mechanism—specifically runaway feedback, network fragmentation, and dissociation across levels—are increasingly supported by contemporary data. For instance, network-oriented work has demonstrated that anxiety and related disorders can be understood as self-sustaining symptom networks, where strongly connected clusters of symptoms maintain each other over time, which is consistent with the idea of pathological positive feedback loops (23, 26, 61, 63). Likewise, studies utilizing dynamical systems approaches and intensive longitudinal data indicate that transitions into and out of psychopathological states may reflect non-linear shifts between attractor states, where small perturbations can have disproportionately large effects when the system’s stability is reduced (122, 123). At the neural level, large-scale connectivity studies in anxiety and trauma-related disorders show concurrent hyperconnectivity in salience/fear circuits and hypoconnectivity in regulatory networks, mirroring the notion of complementary hyper- and hypo-active domains (81, 83, 124).

### 6.2 Comparison of the schismogenesis hypothesis with existing models

Compared with network theory, the schismogenesis model is conceptually aligned—it also views symptoms and processes as mutually interacting nodes—but it adds a cybernetic mechanism that explains why specific subnetworks become self-sustaining and how this results in dissociation between hyper- and hypo-active domains across neural, mental, and social levels (6, 26). In relation to stress–diathesis models, we adopt the central idea that vulnerability and stress interact over time, but we reframe “diathesis” as a limited inhibitory reserve of the system and “stress” as increased activation that, beyond a critical threshold, pushes the system into a schismogenic regime. In this sense, the model offers a more explicit description of the dynamics by which stress–vulnerability interactions unfold (76, 125). Finally, regarding neurocircuitry models of anxiety, which highlight dysregulation of amygdala–prefrontal–hippocampal circuits (34, 40), the schismogenesis framework is compatible yet more encompassing: it embeds circuit-based disruptions within a broader multilevel system, showing how neural imbalances interact with cognitive, affective, lifestyle, and social feedback loops.

The schismogenesis framework resonates strongly with the enactive/pluralistic (3E) paradigm in contemporary psychiatry, which conceptualizes mental disorders as emergent from disturbances in embodied, embedded, and enactive agent–environment interactions, rather than merely from isolated brain dysfunctions. As argued by Enactive Psychiatry, mental illness should be understood as breakdowns in “sense-making” processes that unfold across neural, bodily, and social dimensions (126). In parallel, the schismogenesis model posits that pathological anxiety arises when cybernetic feedback loops become dysregulated, destabilizing the integrated functioning of the brain, mind, body, and social context. This view preserves the core 3E insight of multi-level, context-sensitive embodiment, while providing it with a concrete mechanistic articulation in terms of network dissociation and feedback dysregulation (127). Moreover, the schismogenesis proposal can be seen as a reinvigoration of the classic Biopsychosocial (BPS) Model but rendered in systemic and dynamic terms. Instead of simply listing biological, psychological, and social risk factors, the model articulates a cybernetic mechanism that integrates these domains into a coherent causal chain. In doing so, it provides the kind of integrative backbone that has often been lacking in traditional BPS formulations (19).

This systemic account also aligns with advances from Dynamical Systems Theory (DST) as applied to psychopathology. As recently argued by Marten Scheffer and colleagues (128), psychiatric disorders may represent alternative attractor states (stable but pathological “basins of attraction”) within complex dynamic systems, rather than fixed traits. Under this view, mental health is not a static trait but a dynamic property of system resilience; thus, interventions aim to “flip” the system into healthier attractors (122). The schismogenesis model mirrors this logic: therapeutic interventions targeting dissociation, lifestyle imbalance, or E/I imbalance may function as perturbations that restore systemic integration and shift the system back toward a healthy attractor.

Finally, by integrating biological, psychological, social, and environmental levels through explicit mechanisms and feedback dynamics, the schismogenesis framework embodies a genuine form of Integrative Pluralism in Psychiatry (16, 125, 126). As proponents like C. Gauld have argued, integrative pluralism seeks to reconcile multiple explanatory perspectives—neurobiological, phenomenological, and social—within a shared, coherent framework (127). The schismogenesis model offers precisely that: a synthetic architecture capable of hosting multiple levels of explanation while preserving their interactions, thereby avoiding reductionism yet maintaining explanatory power.

### 6.3 Schismogenesis model informs assessment, diagnosis and treatment planning for ASDs

The schismogenesis model reframes clinical assessment of Anxiety Spectrum Disorders (ASDs) as the identification of hyperactive and hypoactive subsystems across neural, psychological, and social domains, as well as the feedback loops that sustain these dynamics. Instead of merely quantifying symptom severity, assessment focuses on how positive feedback amplifies arousal and how inhibitory mechanisms fail to regulate it, resulting in fragmentation and complementary opposition within the system. This multilevel mapping aligns with principles from cybernetics and complex systems, where disruptions of regulatory feedback produce runaway dynamics (129, 130). By integrating physiological, cognitive, and relational indicators of fragmentation, clinicians obtain a more dynamic and systemic profile of vulnerability and dysregulation in ASDs (131, 132).

From a diagnostic standpoint, the schismogenesis model shifts the focus from categorical labels toward dynamic patterns of system behavior, such as escalating loops of hyperarousal and compensatory shutdown. This perspective helps to explain variability across ASD presentations and clarifies why individuals with similar symptom sets may differ in regulatory capacity, inhibitory reserve, and network fragmentation. Diagnosis thus becomes a formulation of how stress, vulnerability, and feedback patterns interact, rather than a mere symptom checklist (133). The model also accounts for treatment resistance, conceptualizing it as the entrenchment of self-reinforcing loops operating across physiological, mental, and social levels.

Treatment planning informed by schismogenesis emphasizes three coordinated goals: interrupting maladaptive positive feedback, strengthening inhibitory regulation, and promoting reintegration across fragmented subsystems. The model supports multicomponent, simultaneous interventions that target neural regulation, mental processes (e.g., emotion-regulation training), and social patterns (e.g., restructuring interpersonal feedback loops) rather than sequential or single-modality approaches. This aligns with systemic theories that highlight the need to restore regulatory balance and coherence in complex adaptive systems (132, 134).

The schismogenesis model complements existing biomedical, cognitive-behavioral, interpersonal, and trauma frameworks by explaining how dysregulation maintains itself through multi-level feedback loops. Unlike linear biomedical models that localize pathology within the individual, schismogenesis emphasizes interactional and network processes across brain, mind, and social systems (130). Compared with CBT, it maintains the importance of cognitive and behavioral mechanisms but embeds them within broader dynamics of positive and negative feedback (135). It also resonates with trauma and attachment models by framing dissociation and mode-splitting as emergent results of system fragmentation, while adding a cybernetic account of how such fragmentation becomes self-reinforcing (21).

Overall, the schismogenesis model encourages clinicians to conceptualize ASDs not as fixed disorders but as dynamic states arising from breakdowns in regulatory balance between hyper- and hypo-active subsystems. It highlights the importance of detecting system-level patterns—such as escalating positive feedback, impaired inhibitory control, and dissociative fragmentation—that cut across neural, psychological, and social levels. This provides a richer and more actionable framework for understanding clinical complexity and tailoring interventions that restore systemic coherence (131, 132). The model thus offers an integrative, cybernetically informed foundation for comprehensive assessment, diagnosis, and treatment planning.

### 6.4 Limitations

We must acknowledge that the schismogenic framework remains speculative and should be regarded as a heuristic, integrative hypothesis rather than a fully validated theory. We note that current empirical support pertains primarily to component processes (e.g., network connectivity, dissociation, and attractor dynamics) rather than to the entire schismogenesis construct. Furthermore, alternative models—such as purely neurocircuit-based or strictly cognitive-behavioral accounts—may explain substantial portions of ASD pathogenesis without invoking multilevel processes. Consequently, rigorous longitudinal, experimental, and computational modeling work will be required to test the specific predictions of the schismogenesis framework.

## 7 Conclusions

Despite significant advancements in understanding the biological, psychological, and social underpinnings of mental disorders, integrating these diverse perspectives remains challenging (9, 136). While the biopsychosocial model provides a framework for understanding the interplay of these factors, the precise mechanisms are still under investigation (137).

Complex systems, including individuals, exhibit three potential adaptation strategies when perturbed: homeodynamics, homeostasis, and schismogenesis. Anxiety Spectrum Disorders (ASDs) could be characterized as schismogenetic dynamic dysfunctions, marked by reduced mental, social, and cerebral connectivity. This reduction leads to decreased flexibility across these domains, creating self-reinforcing patterns over time (138). Consequently, severe and treatment-resistant ASDs necessitate rapid, personalized, and often multicomponent and synchronous therapeutic interventions.

Systems thinking has been extensively applied in many prominent psychosocial therapeutic approaches for mental disorders, including the concept of schismogenesis in some cases (24, 139–142). However, these systemic therapeutic approaches have not been as frequently extended to the biological realm, and even less so using the construct of schismogenesis isomorphically (143). This study proposes addressing this gap and suggests several hypotheses that warrant further investigation to explore their potential contribution to improving the comprehensive treatment of patients with ASDs.

## Data Availability

The original contributions presented in the study are included in the article/supplementary material. Further inquiries can be directed to the corresponding author.
